# Assessment of knowledge and awareness of monkeypox viral infection in Palestine: a community-based study

**DOI:** 10.3389/fcimb.2025.1584848

**Published:** 2025-08-01

**Authors:** Nuha El Sharif, Muna Ahmead

**Affiliations:** Faculty of Public Health, Al Quds University, Jerusalem, Palestine

**Keywords:** monkeypox, public, knowledge, Palestine, awareness

## Abstract

**Background:**

Monkeypox (MPXV) is a re-emerging global health threat, particularly in non-endemic areas such as Palestine, where research is scarce. This study aims to assess public knowledge and awareness of MPXV in Palestine

**Methods:**

A 38-item questionnaire assessed socioeconomic status, health status, and MPXV knowledge among 1241 participants. Pearson’s Chi-square test examined the association between high and low knowledge levels and explanatory variables. A logistic regression model examined the relationships between knowledge levels and the explanatory factors of the investigation.

**Results:**

The study indicated that merely 23% of the 1241 participants exhibited a high level of knowledge regarding monkeypox. Approximately 20% of the participants felt that the virus might be transmitted through contaminated surfaces, whereas 40% acknowledged close contact with infected individuals as a mode of transmission. Furthermore, 11.4% of the participants accurately identified an incubation period of 5 to 21 days. Concerning symptoms, 50% identified rash and high fever as the most severe, whereas 25% reported fatigue, muscle pain, swollen lymph nodes, and breathing issues. Regarding prevention and treatment, 38% asserted that masks may not prevent MPXV, 31% claimed that no treatment is available, and 50% believed that immunization can be effective. Knowledge was significantly higher among those with more educational attainment and healthcare employment (p-value< 0.05). The primary sources of information were friends, family, and social media.

**Conclusion:**

The understanding of monkeypox infection among the Palestinian population was found to be somewhat insufficient. These findings highlight the urgent need for public education on monkeypox to increase awareness and engage the public prior to any potential future outbreak.

## 1 Introduction

Monkeypox, an enveloped double-stranded DNA virus, is transmitted via zoonotic infection. This virus belongs to the Orthopoxvirus genus within the Poxviridae family (Bunge et al., 2022; Kamal et al., 2023). The incubation period for the monkeypox virus varies from 5 to 21 days. In the first three days, individuals commonly exhibit nonspecific symptoms, including fever and fatigue. Consequently, patients may experience headaches, lymphadenopathy, back pain, and myalgia. The exanthem, or rash, generally appears 2 to 4 days after the onset of fever (Osborn et al., 2022).

The resurgence of international travel following the COVID-19 pandemic has heightened the risk of monkeypox transmission (Adalja and Inglesby, 2022). Due to its swift proliferation and the potential for a pandemic, the World Health Organization (WHO) has elevated its advisory level to the highest alert and declared monkeypox a global public health emergency (United Nations, 2024). As of August 2022, the WHO recorded 39,110 confirmed cases and 12 fatalities across 94 countries (WHO, 2022b). Since then, the World Health Organization (WHO) has recorded instances beyond Africa, in regions where MPXV transmission was previously unknown. By August 2024, the worldwide case total approached 21,000, with an elevated reported mortality rate of approximately 3%. While monkeypox is considered endemic in Africa, the absence of robust surveillance systems makes the true prevalence of the disease unknown (Ihekweazu et al., 2020). In 2022, the United Arab Emirates (UAE) reported 16 cases, while Lebanon, Saudi Arabia, Morocco, and Qatar each reported one case (Farahat and Elsaid, 2022). Jordan also reported a single case (Jordan records first monkeypox case, 2022). Thus, population-based studies are essential in nations where the virus is not yet endemic to evaluate preparedness and guide public health actions.

Recent literature emphasizes that effective outbreak response relies on clinical and virological understanding, as well as public recognition of symptoms, adoption of preventive behaviors, and adherence to public health guidance (Wilson et al., 2014; Petersen et al., 2019b; Huang et al., 2022; Osborn et al., 2022; Soheili et al., 2022). Additionally, public knowledge and awareness are crucial for effective control of the monkeypox disease. This facilitates early symptom recognition, timely medical intervention, and the adoption of preventive measures to limit transmission. Consequently, adhering to appropriate hygiene practices and protocols, such as regular hand hygiene, and minimizing interactions with wildlife, represents critical preventive strategies (Petersen et al., 2019b; Islam et al., 2023). The World Health Organization identifies a lack of information about monkeypox as a significant barrier to preventing its recurrence (WHO, 2017; Harapan et al., 2020). Ultimately, informed communities play a critical role in containing the spread of monkeypox and protecting public health.

While some studies have looked into how much healthcare workers, school students, and university students know about MPXV, these studies have shown that their understanding is often not enough (Harapan et al., 2020; Jairoun et al., 2022; Riccò et al., 2022; Sallam et al., 2022). Furthermore, the public’s awareness of MPXV remains notably limited. There is a significant lack of evidence concerning public awareness of MPXV, particularly in Palestine.

The COVID-19 epidemic in Palestine highlighted the difficulties arising from insufficient understanding of new infectious illnesses. At that time, there was limited data available on the genesis, survival duration, transmission routes, consequences, preventive strategies, and therapeutic options of COVID-19. The virus and effective self-defense strategies were extensively discussed (Hejaz, 2020). Findings from the COVID-19 pandemic demonstrate that disease understanding is crucial for effective prevention (Girometti et al., 2022; Patel et al., 2022; Philpott et al., 2022; United Nations, 2024).

Examining Palestinian population awareness and knowledge regarding MPXV is essential for early preparedness, especially given the virus’s zoonotic nature and expanding global spread. The infectious MPXV virus causes the disease, which has an incubation period of 5 to 21 days before symptoms like fever, rash, lymphadenopathy, myalgia, and occasionally respiratory and oral lesions appear (Islam et al., 2023; Karagoz et al., 2023). Palestine has not reported any cases in the event of an epidemic; detection and response may be delayed due to regional vulnerability and low public awareness. To stop transmission and lessen possible health effects, understanding of knowledge and assessing public awareness can aid in identifying misconceptions, directing focused public health messaging, and promoting prompt, culturally appropriate interventions. Public and healthcare worker awareness is critical to strengthening national preparedness by identifying knowledge gaps, guiding tailored health communication, and training programs. It also supports the development of surveillance systems, response protocols, and infrastructure improvements necessary for timely case management and outbreak control. Consequently, to mitigate the spread of monkeypox within the country, the public must be thoroughly informed about the disease, its associated risks, and preventive measures (Alshahrani et al., 2022).

MPXV has not been investigated in Palestine, especially among the general population. This study aimed to assess people’s knowledge and awareness of monkeypox in Palestine, as there has been limited research on MPXV in the general population. It also sought to examine the association between monkeypox knowledge and sociodemographic factors, sources of information, and health-related variables.

## 2 Methods

### 2.1 Study design and sampling

A descriptive cross-sectional survey was conducted in September 2024. The target population included all Palestinians in the West Bank aged 18 and above. The study expected that 50% of respondents would have sufficient understanding of MPXV to ascertain the required sample size. The minimum sample size determined, utilizing a 95% confidence interval and a 3% margin of error, was 1067 (Daniel and Cross, 2018). Data was collected using an anonymous, online, self-administered survey. Due to Israeli military restrictions on mobility and closures in Palestine, an electronic questionnaire was generated using Enketo Express for the KoboToolbox platform. Participants were recruited via convenience and snowball sampling techniques, primarily through social media channels, including WhatsApp and Facebook Messenger. Participants disseminated the survey link nationwide, and 1241 individuals replied.

### 2.2 Tools and measures

The self-reported questionnaire was developed based on previous research (Alshahrani et al., 2022; Lin et al., 2022; Al-Mustapha et al., 2023). Section 1: This section collected data on age, gender, marital status, education level, employment status, monthly income, healthcare employment, and residential location. Participants were also asked about chronic conditions (hypertension, diabetes, cardiovascular disease, and cancer), prior coronavirus infection, and COVID-19 vaccination status. Section 2: This section consisted of 38 questions assessing the awareness and knowledge of monkeypox, including transmission mechanisms, symptoms, prevention strategies, and care. These questions demonstrated strong internal consistency, with a Cronbach’s α of 0.831.

Two epidemiologists and mental health experts evaluated the cultural relevance and validity of the measure. No alterations to the content or language were required after the pilot study, which involved 20 participants assessing clarity and comprehension. The study team translated the questionnaire into Arabic, which was subsequently back-translated into English by a professional translator. The accuracy of the original English version and the back-translated version was subsequently validated.

### 2.3 Statistical analysis

Data analysis was conducted using SPSS version 25 (IBM Corp., Chicago, Illinois, USA). Descriptive statistics, including frequencies and chi-square tests, were used to analyze quantitative and categorical variables.

For each knowledge question, a score of 1 was awarded for a valid response, while a wrong response or “I don’t know” was granted a score of 0. In questions with multiple correct answers, each correct response received a score of 0.25, culminating in a total score of 1 for the entire question. Total knowledge scores were computed for each participant using the 38-item scale. One of two categories—”low” or “high” MPXV knowledge—was assigned to each participant. Researchers employed a cutoff score of 19 points, which is equivalent to 50% of the maximum achievable score. Participants scoring below 19 points were categorized as possessing “low” knowledge, and those earning 19 to 38 points were categorized as possessing “high” knowledge.

Multivariable logistic regression analyses were performed to assess the relationship between various information sources and awareness variables with MPXV knowledge and to control for potential confounding variables. All significant associations between independent variables and MPXV knowledge were evaluated at a significance level of p < 0.05, with a 95% confidence interval.

### 2.4 Ethical approval and consent to participate

All research methods were conducted following the principles of the Helsinki Declaration. Ethical approval was obtained from the Al-Quds Research Ethics Committee (Ref: 412/REC/2024). Social media data was accessed and analyzed in compliance with the respective platform’s terms of use and all relevant institutional and national regulations. The online survey was anonymous, and participants were provided with written information regarding the study’s purpose and intended data usage at the beginning of the survey. Informed consent was obtained from all participants by completing the questionnaire.

## 3 Results

### 3.1 Respondent characteristics

This online cross-sectional survey included 1241 participants. The demographic profile revealed that approximately 55% were under the age of 35, and 71.4% were female. A majority (66%) were no single, and 45.3% resided in urban areas. Educational attainment was high, with 79.3% having completed more than 12 years of education. In terms of employment, 62.4% were employed, and 28.7% worked in the healthcare sector. Nearly half (48%) reported a monthly income of less than $1080. Regarding health status, approximately 25% reported having chronic diseases, including hypertension, asthma, respiratory problems, cancer, cardiovascular disorders, and diabetes. Additionally, 55.1% of the participants reported a prior coronavirus infection (**Table 1**).

**Table 1:** **C**haracteristics of the study respondents (*N* = 1241).

Variables	Frequency	Percentage
Age in years		
– 18-25	327	26.6%
– 26-35	343	27.9%
– 36-50	413	33.6%
– >51	147	12.0%
Gender		
– male	352	28.6%
– female	878	71.4%
Marital Status		
– Not single	812	66.0%
– single	418	34.0%
Educational level		
– ≤12 years	255	20.7%
– >12	975	79.3%
Place of Residence		
– City	557	45.3%
– Village	510	41.5%
– Refugee camp	163	13.3%
Area of residence		
– North	259	21.1%
– Middle	404	32.8%
– South	567	46.1%
Work now		
– Yes	767	62.4%
– No	463	37.6%
Work in the health sector		
– No	876	71.3%
– Yes	353	28.7%
Monthly income		
– ≤540$	238	19.3%
– 541-1080$	359	29.2%
– 1081-1921$	270	22.0%
– > 1621$	189	15.4%
– No income	174	14.1%
Have chronic diseases		
– Yes	301	24.5%
– No	929	75.5%
Infected with COVID-19		
– No	552	44.9%
– Yes	678	55.1%

### 3.2 Knowledge of MPXV infection

According to the 38-item knowledge measure questions, participants were classified as having “low” or “high” MPXV knowledge. A cutoff score of 19 points, 50% of the maximum score, was applied. 77% of the respondents reported a low knowledge level, and 23% had a high level of knowledge. Accordingly, 81.5% (n = 1142) of respondents had heard of MPXV, and 66.6% correctly identified it as a viral disease. However, only 34.1% (n = 480) believed it was common in the Arab world (**Table 2**). For modes of transmission, a significant proportion (72%) recognized person-to-person transmission. Additionally, 26.2% acknowledged the possibility of asymptomatic MPXV infections, while only 11.4% correctly identified the incubation period as 5–21 days. For how the virus spreads, 20.3% knew it could be transmitted by touching contaminated surfaces, 40% recognized that it spreads through contact with infected people, 11.1% identified hunting and interacting with wildlife as possible ways to catch it, and 6.4% were aware that handling and eating undercooked animal products could also pose a risk. Regarding preventive measures, 51.2% were aware of the existence of a vaccination against MPXV, and 14.2% understood the importance of practicing safe sex to reduce transmission. Conversely, approximately 38% did not believe that wearing masks was an effective preventive measure. In terms of MPXV management, 31% correctly stated that there is currently no specific treatment for the virus, and 24% (n = 338) were aware of the existence of a protective vaccine (**Table 3**).

**Table 2:** Responses to questions assessing participants’ general knowledge about the MPXV virus (Total 1241).

Variables	Responses	Frequency	Percentage
Have you heard of the MPXV outbreak?	No	229	18.5%
	Yes	1012	81.5%
MPXV is a viral disease	No	32	2.6%
	Yes	827	66.6%
	Don’t know	382	30.8%
MPXV can be transmitted from one person to person	No	29	2.3%
	Yes	894	72.0%
	Don’t know	318	25.6%
Are you aware that the MPXV was declared a global health emergency?	No	259	20.9%
	Yes	636	51.2%
	Don’t know	346	27.9%
MPXV, present in some parts of the Arab world, is common	No	243	19.6%
	Yes	423	34.1%
	Don’t know	575	46.3%
Can we have asymptomatic cases of the MPXV?	No	179	14.4%
	Yes	325	26.2%
	Don’t know	737	59.4%
Anyone can be infected	True	716	57.7%
– Teenagers	True	17	1.4%
– People with chronic diseases	True	47	3.8%
– Only those who practice unsafe sex	True	25	2.0%
– Only old people	True	26	2.1%
Which of these can be protective against MPXV?			
– Antibiotics (False)	False	1062	85.5%
– Proper hygiene, such as handwashing	True	793	63.9%
– Practicing safe sex	True	176	14.2%
– Vaccination against	True	636	51.2%
How important is hand hygiene in reducing the spread of viruses (MPXV virus)?	Not important	30	2.4%
	Averagely important	175	14.1%
	Extremely important	1036	83.5%
Washing hands and wearing masks are effective means to prevent MPXV	False	71	5.7%
	True	764	61.6%
	Don’t know	406	32.7%
Healthcare workers are at a greater risk of MPXV infection	False	78	6.3%
	True	797	64.2%
	Don’t know	366	29.5%

**Table 3:** Responses to questions assessing participants’ knowledge about the MPXV virus signs, symptoms, and treatment (Total 1241).

Variables	Responses	Frequency	Percentage
What is the incubation period for MPXV?	Don’t know	837	67.4%
	1–14 day	242	19.5%
	5–21 day	142	11.4%
	1–3 months	20	1.6%
Are you aware of MPXV virus symptoms?			
– Swollen lymph nodes	True	285	23.0%
– High fever	True	639	51.5%
– Fatigue	True	356	28.7%
– Rashes	True	646	52.1%
– Sores in the mouth, vagina, hands, anus	True	252	20.3%
– Muscle pain	True	334	26.9%
– Runny nose	True	148	11.9%
– Dry cough	True	267	21.5%
– Breathing difficulties	True	135	10.9%
– Bleeding	True	37	3.0%
– Diarrhea	False	1090	87.8%
– Hair loss	False	1215	97.9%
How does the virus spread?			
– Air droplets	False	1119	90.1%
– Mosquito/Insect bites	False	1074	86.5%
– Sexual intercourse	False	1047	84.3%
– Contact with contaminated surfaces	True	252	20.3%
– Close contact with people who have the virus	True	496	40.0%
– Hunting and contact with wildlife	True	138	11.1%
– Processing & consumption of inadequately cooked animal products	True	79	6.4%
There are currently no specific treatments for MPXV infections	False	108	8.7%
	True	385	31.0%
	Don’t know	748	60.3%
There is a vaccine that protects against MPXV	False	133	10.7%
	True	305	24.6%
	Don’t know	803	64.7%
MPXV is usually self-limiting, with the symptoms lasting from 2 to 4 weeks.	False	120	9.7%
	True	286	23.0%
	Don’t know	835	67.3%
We can use herbs to recover from the MPXV since it is a flu	False	321	25.9%
	True	107	8.6%
	Don’t know	813	65.5%

**Figure 1:**
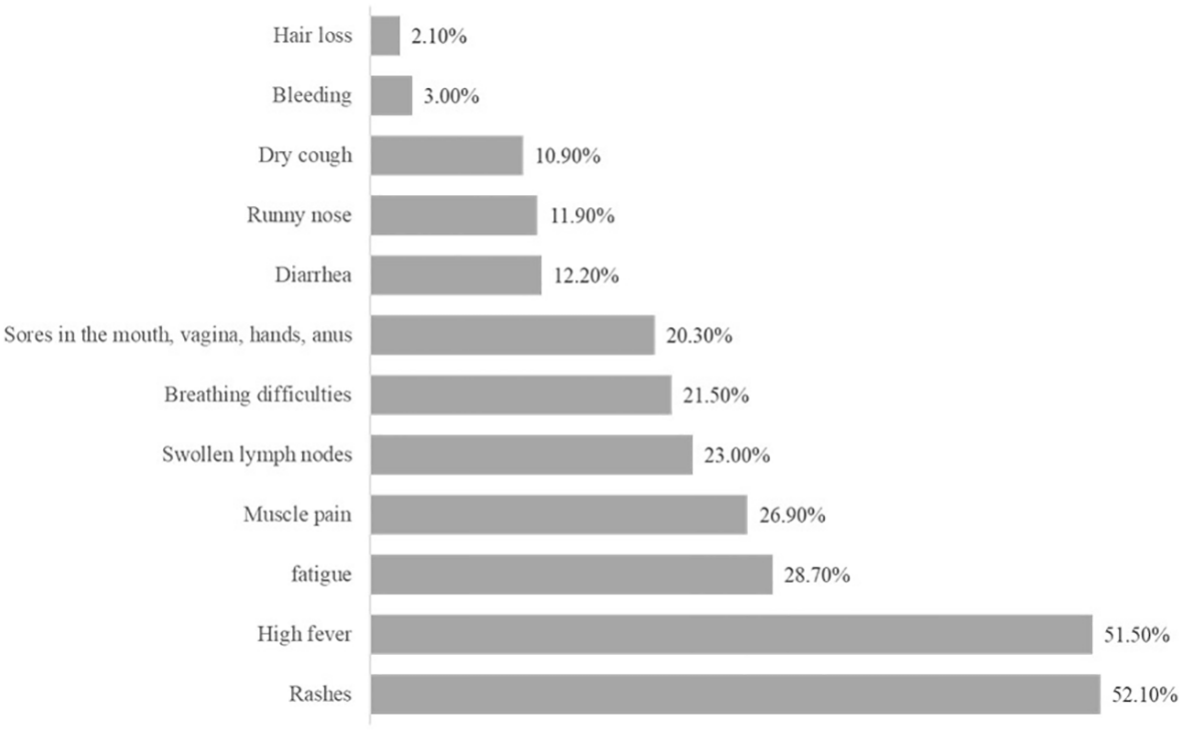
Source of information on MPXV.

Concerning awareness of signs and symptoms, approximately half of the participants recognized rash and high fever as the most significant symptoms of MPXV. About 25% were also aware of other common symptoms, including fatigue, muscle pain, swollen lymph nodes, respiratory problems, and oral sores. The primary sources of information about MPXV were digital platforms. Specifically, 49.8% of respondents reported obtaining information from internet sources, and 40.9% cited social media platforms as their source (**Figure 1**).

### 3.3 Association between the personal factors, awareness, and knowledge of MPXV disease among study participants

The study revealed that 23% of participants demonstrated high knowledge of human monkeypox, while 77% exhibited low knowledge. When examining the association between knowledge levels and sociodemographic and health factors (**Table 4**), several variables showed significant associations (p < 0.05). These included participant age, education level, place of residence, employment status, income level, healthcare employment, and prior coronavirus infection. However, no significant associations were found with participant gender, marital status, chronic disease status, or prior COVID-19 vaccination (p > 0.05).

**Table 4:** Association between the knowledge score and the socio-demographics among respondents (n = 1241).

Variables		Low N=955		High N=286		Chi-square P value
		n	%	n	N %	
Age in years	18-25	277	29.0%	51	17.8%	<0.001
	26-35	244	25.5%	100	35.0%	
	36-50	317	33.2%	102	35.7%	
	>51	117	12.3%	33	11.5%	
Gender	Male	275	28.8%	81	28.3%	0.86
	Female	680	71.2%	205	71.7%	
Marital Status	Not single	624	65.3%	198	69.2%	0.22
	Single	331	34.7%	88	30.8%	
Educational level	≤12 years	226	23.7%	38	13.3%	<0.001
	>12	729	76.3%	248	86.7%	
Place of residence	City	394	41.3%	165	57.7%	<0.001
	village	415	43.5%	95	33.2%	
	Refugee camp	146	15.3%	26	9.1%	
Area of residence	North	202	21.2%	57	19.9%	0.06
	Middle	325	34.0%	79	27.6%	
	South	428	44.8%	150	52.4%	
Working now	yes	553	57.9%	219	76.6%	<0.001
	no	402	42.1%	67	23.4%	
Monthly income level	≤540$	202	21.2%	39	13.6%	<0.001
	541-1080$	293	30.7%	72	25.2%	
	1081-1921$	189	19.8%	83	29.0%	
	> 1621$	122	12.8%	67	23.4%	
	No income	149	15.6%	25	8.7%	
Have a chronic disease	Yes	242	25.3%	66	23.1%	0.44
	No	713	74.7%	220	76.9%	
Get infected with COVID-19	No	452	47.3%	104	36.4%	0.001
	Yes	503	52.7%	182	63.6%	
Work in the health sector	No	759	79.5%	129	45.1%	<0.001
	Yes	196	20.5%	157	54.9%	
Had the COVID-19 vaccine	No	138	14.5%	30	10.5%	0.08
	Yes	817	85.5%	256	89.5%	

### 3.4 Multivariate analysis

In the multivariate analysis (**Table 5**), those with over 12 years of education exhibited a higher likelihood of having high knowledge compared to those with 12 years of education or less (AOR=1.78, p-value=0.009). Furthermore, residents of the southern areas of the West Bank were more likely to have higher knowledge compared to those living in other locations. In addition, individuals employed in the health sector had about 3.5 times higher likelihood of knowledge of MPXV compared to other participants. Finally, the sources of information that increased the likelihood of having a high level of knowledge about MPXV by two to three times were television (TV), the internet, social media platforms, the WHO, and material provided by healthcare professionals.

**Table 5:** Multivariate analysis models of participants’ characteristics and awareness of participants and knowledge about MPXV.

Variables	Sig.	AOR	95% CI.for AOR	
			Lower	Upper
Age in years				
– 18-25	0.10	0.62	0.34	1.11
– 26-35	0.30	1.32	0.77	2.25
– 36-50	0.79	1.07	0.63	1.80
– >51		Reference		
Educational level				
– >12 years	0.009	1.78	1.15	2.77
– ≤12		Reference		
Area of residence	0.003			
– North	0.48	0.86	0.57	1.30
– Middle	0.001	0.53	0.36	0.76
– South		Reference		
Place of Residence	0.011			
– City	0.10	1.54	0.90	2.62
– village	0.81	0.93	0.5	1.62
– Refugee camp		Reference		
Monthly income	0.01			
– ≤540$	0.92	1.03	0.55	1.91
– 541-1080$	0.69	0.89	0.49	1.59
– 1081-1921$	0.23	1.43	0.79	2.59
– > 1621$	0.03	1.92	1.03	3.55
– No income		Reference		
Work in the health sector (Yes/No)	<0.001	3.52	2.52	4.90
Source of knowledge				
– Television (Yes/No)	<0.001	1.98	1.38	2.86
– Internet sources (Yes/No)	0.001	1.70	1.25	2.33
– Social media platforms (Yes/No)	<0.001	2.07	1.51	2.83
– WHO website (Yes/No)	<0.001	2.34	1.53	3.59
– Friends/Family (Yes/No)	.000	4.64	2.89	7.46

AOR, Adjusted odds ratio; 95% CI, 95% confidence interval; sig., significance.

The logistic regression model includes all personal characteristics (participants’ age, gender, marital status, educational level, residence location, area of residence, work status, chronic diseases, previously infected with COVID-19, and working in the health sector) and source of knowledge (TV, Radio, internet sources, social media platforms, newspaper, friends/family, WHO website, books, healthcare provider.

## 4 Discussion

Given the rising occurrence of monkeypox in non-endemic nations, it is essential for the Palestinian government and population to be equipped for outbreak prevention. Although public health and healthcare officials are responsible for formulating strategies for epidemic prevention, control, and treatment, public engagement is essential for the efficacy of these activities. This study represents the first preliminary investigation of public awareness and knowledge of monkeypox in Palestine.

The findings of our study revealed that fewer than one-quarter of the respondents possessed high knowledge about monkeypox infection. This outcome is significantly lower than research conducted in nations experiencing occasional outbreaks. Public knowledge was reported as 48% in Saudi Arabia, 58.7% in Nigeria, and 32.9% in Yemen (Halboup et al., 2023). A systematic review examining MPXV knowledge across multiple studies revealed that a significant proportion (53.4%) of participants exhibited insufficient understanding (Tanashat et al., 2024).

The study revealed that 50% of participants identified MPXV as a global health concern, whereas only 25% recognized its prevalence in the Arab region. Significantly, no instances of MPXV have been recorded in Palestine. Despite recent discourse regarding MPXV symptoms and transmission pathways on local radio and television, it is not regarded as an urgent issue within the Palestinian population. The levels of awareness are superior to those documented in certain other investigations (Alshahrani et al., 2022; Al-Mustapha et al., 2023; Youssef et al., 2023; Das et al., 2024).

Approximately two-thirds of the Saudi population were aware of the recent emergence of monkeypox in other countries. However, a majority perceived monkeypox as rare in the Middle Eastern region, and nearly three-quarters were unaware of any cases within Saudi Arabia (Alshahrani et al., 2022). In contrast, in Nigeria, 89% of respondents were aware of the 2022 monkeypox outbreak, while 17% recognized its designation as a Public Health Emergency of International Concern (PHEIC). Given that monkeypox is endemic to Nigeria, their experience with the virus, coupled with higher educational levels among respondents and numerous online public health notifications, likely contributed to enhanced public knowledge of the emerging epidemic (Al-Mustapha et al., 2023). Therefore, the public health authorities in Palestine should strengthen risk communication using culturally tailored messages through trusted media channels. Community education and targeted outreach can raise awareness without causing alarm, ensuring the population remains informed and prepared.

Understanding the signs and symptoms of MPXV is critical to comprehending the virus. In this study, approximately 25% of respondents believed that MPXV-infected individuals could be asymptomatic, while 11.4% were aware of the virus’s incubation period of 5 to 21 days. Roughly 50% of respondents identified rash and elevated temperature as key symptoms, and 25% recognized fatigue, myalgia, lymphadenopathy, respiratory distress, and oral lesions as other common symptoms. It’s important to note that symptom awareness varies across different populations. Generally, human monkeypox symptoms are self-limiting, typically resolving within 14 to 21 days (Wilson et al., 2014; Guzzetta et al., 2022; Huang et al., 2022; CDC, 2024) with an incubation period ranging from 5 to 21 days (Altindis et al., 2022; Guzzetta et al., 2022). A study in the UAE indicated a 56% optimal awareness level for MPXV transmission and symptoms (Zeidan et al., 2023). In Yemen, 34.9% of respondents acknowledged flu-like symptoms as a possible early sign of MPXV, whereas 63% recognized skin rash and vesicles as characteristic symptoms. In Saudi Arabia, 80% of respondents recognized skin rash as a symptom, although only 53.8% acknowledged the presence of flu-like symptoms (Halboup et al., 2023). The overall clinical presentation of monkeypox resembles that of smallpox, but with less severity (Petersen et al., 2019a).Symptoms include pruritic or painful dermal lesions, fever, generalized headache, fatigue, lymphadenopathy, back pain, and muscle aches (Wilson et al., 2014; Huang et al., 2022). Skin rash, appearing within three days of fever resolution, is the most prominent clinical symptom. This rash typically begins on the face and spreads centrifugally throughout the body, including the extremities. In severe cases, it may cover the entire body (Osborn et al., 2022; Soheili et al., 2022). The respondents’ potential lack of awareness regarding MPXV symptoms, causes, transmission, treatment, and care may stem from insufficient information dissemination. The low public awareness of monkeypox symptoms, transmission, and severity, as highlighted by this study, poses a significant challenge to future surveillance efforts in Palestine and similar settings. According to the Health Belief Model, when individuals do not perceive themselves to be at risk or lack knowledge about the disease, they are less likely to seek care, report symptoms, or adhere to preventive measures (Karl et al., 2022). Additionally, limited mpox knowledge may lead to low self-efficacy to carry out preventive measures to protect themselves (Hassan et al., 2024).

Due to limited reporting in the Middle East, particularly in Arab nations, the disease remains poorly understood. A deficiency in public engagement may also contribute to this knowledge gap. Furthermore, local political instability has diverted attention from global health concerns, focusing instead on domestic affairs. Consequently, MPXV could spread and be diagnosed belatedly, posing a significant public health risk. The healthcare system may be inadequately prepared at the onset of an epidemic. Therefore, Palestinian health authorities should implement targeted health education campaigns that clearly communicate the full spectrum of MPXV symptoms and the potential for asymptomatic transmission. These campaigns should use accessible language and trusted communication channels such as primary care centers, schools, media, and community leaders. Strengthening public health communication and community-level awareness is therefore essential to support effective surveillance and early outbreak response systems for monkeypox.

Despite the absence of reported monkeypox cases in Palestine, our study revealed that a significant proportion (72%) of participants believed the MPXV virus is transmitted through human contact. Monkeypox spreads from one person to another through close contact with someone who is infected, which can include being near them when they breathe, touching their skin sores, having genital contact, spending a lot of time face-to-face, or coming into contact with dirty bedding and clothes. Initial research suggested that MPXV exhibits a lower probability of human-to-human transmission compared to smallpox (Yinka-Ogunleye et al., 2019).

Furthermore, only 17.2% of participants identified more than three sources of transmission, and only 6% recognized at least one (data not shown). Specifically, 20.3% were aware of transmission through contact with contaminated surfaces, and 40% recognized transmission through contact with infected individuals. These results indicate that Palestinian knowledge of MPXV transmission is considerably lower than in African countries where the virus is endemic or where significant case numbers have been reported (Yinka-Ogunleye et al., 2019; Diatta et al., 2023). In Pakistan, 85.69% of the general population stated that a virus causes MPXV, and about 72.18% believed it spreads through contaminated surfaces. Our findings align with studies conducted in non-endemic countries (Yousaf et al., 2025). In Yemen, individuals reported fear of visiting friends or family or traveling due to concerns about MPXV transmission (Halboup et al., 2023). Our findings reveal significant deficiencies in risk perception, a fundamental component of effective public health response, which remain alarmingly low within the surveyed population. This indicates a profound misunderstanding of the virus’s transmission dynamics, substantially impeding individuals’ capacity to accurately evaluate their risk (Joslyn et al., 2021; Crosato et al., 2024). A comprehensive understanding of all transmission pathways, including less prevalent yet pertinent ones such as respiratory droplets, is essential for an exhaustive risk evaluation and the subsequent implementation of appropriate protective measures. Public health continues to place a high premium on ensuring that the general public is well-informed, comprehensive, and useful (Joslyn et al., 2021; Crosato et al., 2024). Consequently, enhancing public knowledge and awareness is crucial to prevent the global spread of the disease.

Regarding preventive measures, 50% of participants were aware of the MPXV vaccination, and a majority recognized that hand hygiene can mitigate virus transmission. However, only 14.2% believed that safe sex was essential for preventing MPXV and managing the outbreak, and 38% did not consider masks to be effective. The general understanding of hand hygiene’s role in mitigating viral infections likely stems from experiences during the COVID-19 outbreak. While half of the participants were aware of the vaccine, some believed it was not protective. The finding is significant because, according to the Health Belief Model, MPXV’s acceptability and uptake of vaccinations may imply higher perceived susceptibility and severity of the illness, which may motivate individuals to modify their behaviors (Nadarzynski et al., 2021).

Approximately 70% of participants reported obtaining information through social media and the internet (data not shown). This points out the potential for misinformation dissemination through these channels, demonstrating the importance of using reliable sources to educate the public about MPXV infection, transmission, and preventive measures. Studies have demonstrated that easy internet access enhances public access to health information (Lee et al., 2021). However, the WHO has identified insufficient disease understanding as a significant obstacle in preventing monkeypox re-emergence (WHO, 2022a).

To combat the spread of misinformation and fallacies online, particularly concerning epidemic treatments and hazardous alternatives, the WHO has developed “Questions and Answers” resources. Monkeypox narratives often include misconceptions about sexual transmission, exclusive transmission from monkeys, conspiracy theories, clandestine therapies, and laboratory origins. Therefore, education on viral transmission should emphasize the importance of accurate virus knowledge, prevention, and prompt screening and identification. The COVID-19 outbreak in Palestine demonstrated that traditional media (television, radio, newspapers) can mitigate the spread of conspiracy theories and disinformation, whereas digital media and interpersonal interactions can exacerbate them. Misinformation regarding COVID-19 was also shown to induce anxiety (Maraqa et al., 2020).

Our logistic regression analysis revealed a significant association between high education, residence in the southern West Bank, healthcare employment, and greater awareness of monkeypox. These findings align with previous studies that demonstrated strong correlations between sociodemographic variables and infectious disease awareness, suggesting that education and occupation play critical roles in health knowledge acquisition (Alshahrani and Sah, 2022; Halboup et al., 2023; Malaeb et al., 2023; Yousaf et al., 2025). For example, in Armenia, younger age, being a female, high level of education, city residence, and being employed were associated with higher mean scores of infectious disease-related health literacy and knowledge (Sargsyan et al., 2024). Whereas in Lebanon, being a female, increased age, and living in rural areas were found to be negatively associated with a good level of MPXV knowledge (Youssef et al., 2023).

In addition, our results indicated that healthcare employment was significantly associated with MPXV knowledge. This finding was expected, as healthcare professionals tend to be younger and more educated. These groups are likely to have access to credible information, engage with scientific literature, and possess the ability to critically evaluate information sources. Also, they are more likely to engage with institutional health updates and clinical training, which enhances their knowledge of emerging infectious diseases such as monkeypox (Al Meslamani et al., 2025)Similar results were observed in recent surveys conducted in Saudi Arabia (Alshahrani et al., 2022), Lebanon (Malaeb et al., 2023), and Yemen (Halboup et al., 2023), whereas individuals with higher education or healthcare employment demonstrated superior knowledge scores. Also, a study in Nigeria indicated that occupation, educational attainment, and age significantly influence MPXV knowledge (Al-Mustapha et al., 2023). However, these associations likely reflect structural and informational inequalities, where access to formal education and urban healthcare infrastructure facilitates exposure to accurate health information (Ratzan et al., 2020). Additionally, younger age, often correlated with recent academic exposure and digital literacy, may also contribute to better engagement with public health campaigns and online health information platforms (Wang et al., 2019). However, while these findings suggest a strong association with monkeypox knowledge, caution is needed in interpretation, as confounding variables such as personal interest in health or differential access to digital resources may also play a role.

Following recommendations from various studies, there is a critical need for close monitoring of this disease, the establishment of appropriate protocols for early detection, and the development of effective prevention and treatment alternatives (Beer and Rao, 2019). Furthermore, primary prevention public awareness campaigns should focus on the disease’s risk factors and the preventive measures that can mitigate those risks (Huang et al., 2022; Jairoun et al., 2022; Lin et al., 2022).

## 5 Study limitations and strengths

This is the first public study in Palestine that investigated monkeypox. We successfully conducted a community-based study that showcases resilience and adaptability in conducting vital research under resource-constrained conditions, establishing a valuable baseline for future studies and highlighting the importance of community-based approaches in challenging settings. Although this study reached a significant number of Palestinians in the West Bank, it has certain limitations. Participation was restricted to internet users, potentially introducing bias because knowledge levels may differ among individuals without internet access. Additionally, the Israeli military incursion in the northern West Bank hindered some individuals from completing the questionnaire. The self-administered nature of the online survey also presents a potential limitation. Furthermore, the use of convenience and snowball sampling techniques may limit the generalizability of the findings. Both sampling methods may lead to homogeneity in the sample; therefore, they might not represent the diversity of the targeted community, thereby constraining external validity. Finally, the study relied on a questionnaire to gather quantitative data, which may not capture the full complexity of public knowledge and attitudes.

## 6 Conclusion

Palestinians revealed a significant knowledge gap regarding the signs, prevention, and treatment of MPXV in the current study. Consequently, enhancing public education regarding monkeypox management, prevention, and treatment is crucial. To achieve this, a platform for disseminating reliable information is necessary to counter the risks posed by misinformation and conspiracy theories. An educational initiative and an awareness program aimed at addressing information deficiencies and correcting misconceptions are essential for effective epidemic preparedness and response at educational settings, i.e., schools and universities. For instance, implementing a health education curriculum that includes modules on emerging infectious diseases such as MPXV is crucial. A “train-the-trainer” program initiated by the Ministry of Health, in cooperation with other healthcare providers such as the UNRWA and other non-governmental organizations, would provide healthcare teams with clinical expertise and communication skills. This training will provide health teams with accessible reference materials for discussing symptoms and prevention during patient consultations. For future crises, set up a strategic plan at the national level to deal with any outbreaks, including medical supplies and well-trained professionals. Ultimately, future research on human MPXV should acknowledge and address the limitations identified in this study.

## Data Availability

The raw data supporting the conclusions of this article will be made available by the authors, without undue reservation.
